# Unimolecular Double Photoionization-Induced Processes in Iron Pentacarbonyl

**DOI:** 10.1021/acs.inorgchem.1c02533

**Published:** 2021-10-26

**Authors:** Roberto Linguerri, Emelie Olsson, Gunnar Nyman, Majdi Hochlaf, John H. D. Eland, Raimund Feifel

**Affiliations:** † COSYS/LISIS, Université Gustave Eiffel, 5 Bd Descartes, 77454, Champs sur Marne, France; ‡ Department of Physics, University of Gothenburg, Origovägen 6B, 412 58 Gothenburg, Sweden; § Department of Chemistry and Molecular Biology, University of Gothenburg, 405 30 Gothenburg, Sweden; ∥ Department of Chemistry, Physical and Theoretical Chemistry Laboratory, Oxford University, South Parks Road, OX1 3QZ Oxford, U.K.

## Abstract

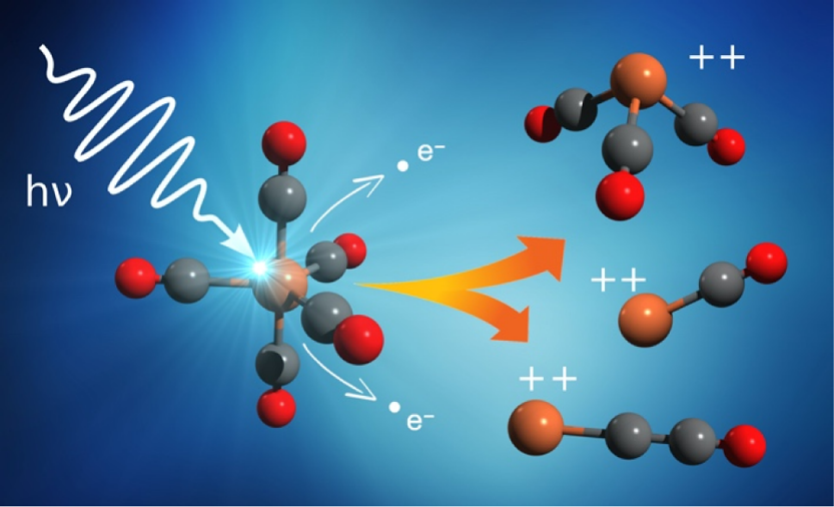

The dissociations of nascent Fe(CO)_5_^++^ ions created by 40.81 eV photoionization of iron pentacarbonyl have been examined using threefold and fourfold electron–ion coincidence measurements. The energies and forms of the ions have been explored by high-level calculations, revealing several new structures. The most stable form of Fe(CO)_5_^++^ has a quite different geometry from that of the neutral molecule. The dissociation pattern can be modeled as a sequence of CO evaporations followed by two-body charge separations. Each Fe(CO)_*n*_^++^ (*n* = 1–4) dication is stable in a restricted energy range; as its internal energy increases, it first ejects a neutral CO, then loses CO^+^ by charge separation at higher energy. In the initial stages, charge-retaining CO evaporations dominate over charge separation, but the latter become more competitive as the number of residual CO ligands decreases. At energies where ionization is mainly from the CO ligands, new Fe–C and C–C bonds are created by a mechanism which might be relevant to catalysis by Fe.

## Abstract 2

Dissociations of nascent Fe(CO)_5_^++^ ions by sequential CO evaporations, leading (in restricted energy ranges) to stable Fe(CO)_*n*_^++^ (*n* = 1−4) dicationic species. At energies where ionization is mainly from the CO ligands, new Fe−C and C−C bonds are created by a mechanism which might be relevant to catalysis by Fe.

## Introduction

Transition-metal ions in the gas phase are highly reactive chemical species, often characterized by complex reaction dynamics, an in-depth understanding of which defies both theory and experiment. These ions are capable, for instance, of breaking bonds in organic compounds such as the C–C bonds of hydrocarbons by means of metal insertion reaction mechanisms^1^ or, in the case of iron carbonyl or naked iron cluster cations, these ions are capable of promoting C–C bond forming reactions.^2−4^ Iron pentacarbonyl is the most stable complex of those with the general formula Fe_*m*_(CO)_*n*_. The equilibrium structure of the neutral molecule is a trigonal bipyramid of *D*_3*h*_ symmetry, where an interesting equatorial-axial ligand scrambling takes place through Berry pseudo-rotations.^5^ The formation and fragmentation of positively charged molecular ions obtained by single or multiple ionization of neutral homoleptic complexes like Fe(CO)_5_ are of considerable interest because of the unusual ease with which the CO ligands can move within the molecule or be lost. Experimental studies of CO ligand losses in transition-metal carbonyl cations are not straightforward but can be accomplished by several techniques, such as the threefold and fourfold electron–ion coincidence or threshold collision-induced dissociation spectroscopies. Such experiments are useful as providing thermochemical data for these species and also as a fundamental tool giving insights into the complex intramolecular and fragmentation dynamics of these ions. One such technique has recently been applied, for example, to the investigation of the dissociative photoionization of the chromium hexacarbonyl complex^6^ for which a revised value of the formation enthalpy has been proposed.

When neutral Fe(CO)_5_ is electronically or otherwise excited, it rapidly evaporates one or more CO molecules, leaving behind exotic iron-containing fragments down as far as bare Fe atoms. The details of these processes have been characterized in depth, particularly by ultrafast pump–probe experiments^7−16^ and related theory.^17^ After some forms of excitation, the neutral molecule loses one CO ligand within 100 fs and a second within 3.3 ps.^8,10^ In a time-resolved study of neutral Fe(CO)_5_ in ethanol, Werner et al.^12^ point out that the charge density at the Fe atom is an important parameter affecting the catalytic properties of the molecule in different states. Ionization, which at low energy occurs predominantly from the Fe 3d orbitals, may therefore provide a benchmark in this regard. In a related study, the sequential nature of multiple CO losses from excited neutral Fe(CO)_5_ in the gas phase was demonstrated directly.^14^ In single ionization of Fe(CO)_5_, the dissociation pathways and their energy dependence and kinematics have been characterized by electron impact,^18,19^ ion impact,^20^ and photon impact^21^ mass-spectrometry and by an electron–ion coincidence technique.^22^ In the singly ionized species, as in neutral molecules, the dominant process is successive loss of neutral CO fragments from the molecule. A similar pattern is expected and has been found for the effects of double ionization of Fe(CO)_5_,^18−20,23^ which forms the subject of this paper, but much less is yet known of the energies or molecular structures involved.

The chemical pathways for decay of nascent [Fe(CO)_5_]^++^ have been studied by electron, ion, and photon impact mass spectrometry^18−20,24^ and by ion–ion coincidence measurements,^25^ and the overall spectrum of doubly positive states formed by photoionization has been measured by electron–electron coincidence spectroscopy.^26^ In the experimental part of the present work, we investigate the energetics of the dissociation processes by threefold (electron–electron–ion) and fourfold (electron–electron–ion–ion) coincidence measurements following photoionization at 40.81 eV photon energy. In the theoretical part, we explore the stabilities and structures of the major dicationic species using the most advanced available computational methods. Theoretical examination of the reaction dynamics is somewhat restricted in this case, due to the great multiplicity of states and structures of the molecule and its fragments, together with the large number of atoms and the fluxional nature of the molecule at normal temperatures.^5^

### Experimental Methods

The apparatus and experimental techniques used in this work have been described in detail before.^27,28^ Briefly, ionization occurs where pulsed monochromatic light from a fast discharge in low-pressure He impinges on molecules in an effusive jet. The ionization zone is embedded in a divergent magnetic field provided by a ring magnet and shaped pole piece, which direct all photoelectrons into a long solenoid whose field guides them to a 2 m distant detector. After all relevant electrons have left the ionization zone, a pulsed draw-out field accelerates positive ions in the opposite direction, through the ring magnet, to a time-of-flight mass spectrometer. Electron and ion arrivals at their respective detectors are timed with nanosecond precision relative to the time of each light pulse. For these experiments, the charged particle count rates were kept to a few hundred per second or fewer compared with a typical light pulse repetition rate of 7 kHz to suppress accidental coincidences to a level where they could be accurately subtracted from the accumulated coincidence signals. The combined collection and detection efficiencies were about 50% for electrons and 10% for ions. The energy resolution for electrons was about 2%, and mass resolution in these experiments was about 5%, sufficient to isolate all the major species but not to measure the abundances of ions containing ^13^C or the minor Fe isotopes separately. Iron pentacarbonyl was a commercial sample used without further purification.

### Theoretical Methods

All electronic structure computations were performed using the MOLPRO suite of programs,^29^ in the C_1_ point group. For neutral and doubly (singly) charged molecular systems of interest for this study, we mapped the lowest singlet and triplet (doublet) potential energy surfaces to find the stable structures. Thus, we carried out coupled cluster [(R)CCSD(T)]^30−33^ and (Restricted) Møller–Plesset to second-order (R)MP2^34^ geometry optimizations (without constraints), where relativistic corrections were introduced at the level of the Douglas–Kroll–Hess (DKH) Hamiltonian. The aug-cc-pVDZ-DK basis set was used throughout all these computations.^35^ The natures of the calculated stationary points on the PESs of these molecules and ions were verified by inspection of their harmonic vibrational frequencies. For true minima, all harmonic frequencies are real.

To investigate the electronic excited states of iron-containing ions of interest, we performed complete active space self-consistent field (CASSCF) computations,^36,37^ including scalar relativistic effects evaluated at the level of the DKH Hamiltonian,^38,39^ together with the aug-cc-pVDZ-DK basis set. Therefore, we employed an active space of 12 orbitals within the valence shell, in which all the possible excitations of 12 electrons were considered. This resulted in around 227 000 and 382 000 configuration state functions (CSFs) for the singlet and triplet states, respectively.

The deduced energetics (e.g., ionization and fragmentation energies and excitation energies) of the Fe(CO)_5_^++^ molecular system are expected to be accurate within 0.1 eV.

### Experimental Results

**Figure 1:**
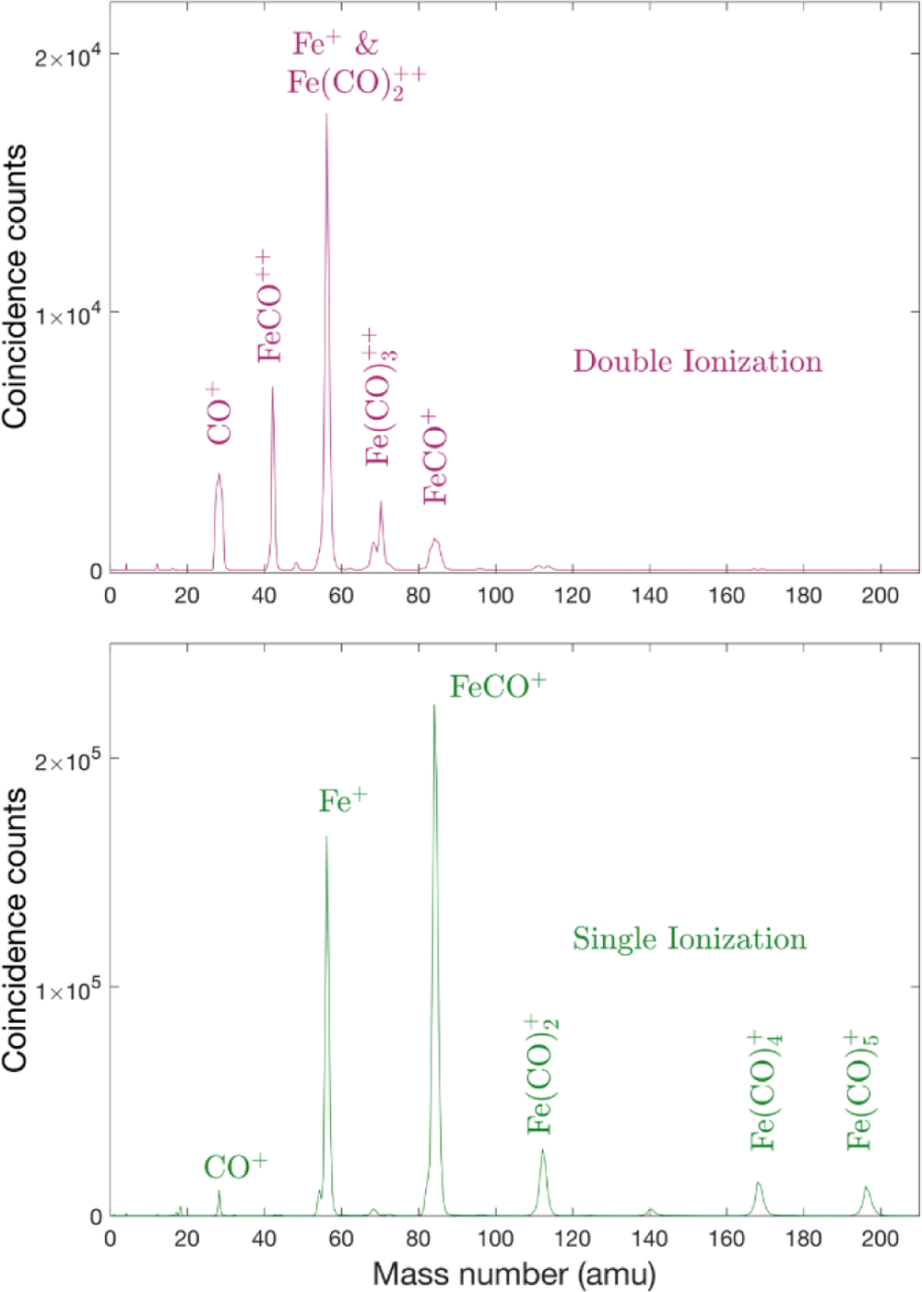
Complete mass spectra from pure single and pure double ionization of Fe(CO)_5_ at 40.81 eV photon energy.

The mass spectrometric examination of iron pentacarbonyl presents a particular difficulty that the main isotope of iron, ^56^Fe, has just twice the mass of the principal light fragment, CO. Thus, the pairs of species Fe^+^ with Fe(CO)_2_^++^ and FeCO^+^ with Fe(CO)_4_^++^ appear at identical mass-to-charge ratios and are indistinguishable without very high mass resolution. The second isotope, ^54^Fe, is not abundant enough to help separate these pairs in our apparatus. Our ability to detect one or two electrons formed in coincidence with each ion provides, however, the unique ability to separate the products from single and double ionization. As a first step, we can obtain separate mass spectra from the two degrees of ionization, as shown in Figure 1.

The mass spectrum from double ionization in Figure 1 contains both doubly charged fragments from charge-retaining decays and singly charged ions from charge separations. Because of the large (3–5 eV) kinetic energy release (KER) from Coulomb repulsion in charge separations, the time-of-flight peaks for charge-separated monocations are distinctly broader than those for doubly charged species such as FeCO^++^ or Fe(CO)_3_^++^. Thus, the shape of the *m*/*z* 84 peak for FeCO^+^ from double photoionization indicates that it is hardly contaminated at all by overlay of any Fe(CO)_4_^++^ dication. The peak for ^56^Fe^+^ at *m*/*z* 56, in contrast, appears likely to have a strong contribution from Fe(CO)_2_^++^ ions. The CO^+^ peak at *m*/*z* 28 does not seem to have a significant contribution from ^56^Fe^++^, so we assume that formation of this ion is at most a minor pathway. The parent doubly charged ion Fe(CO)_5_^++^ is not detectable here (at *m*/*z* 98), but its existence as a stable entity is attested in electron impact mass spectrometry.^19^ Of the doubly charged species observed here [Fe(CO)_3_^++^, Fe(CO)_2_^++^, and FeCO^++^], only FeCO^++^ was observed in the ion impact experiments of Indrajith et al.^20^

**Figure 2:**
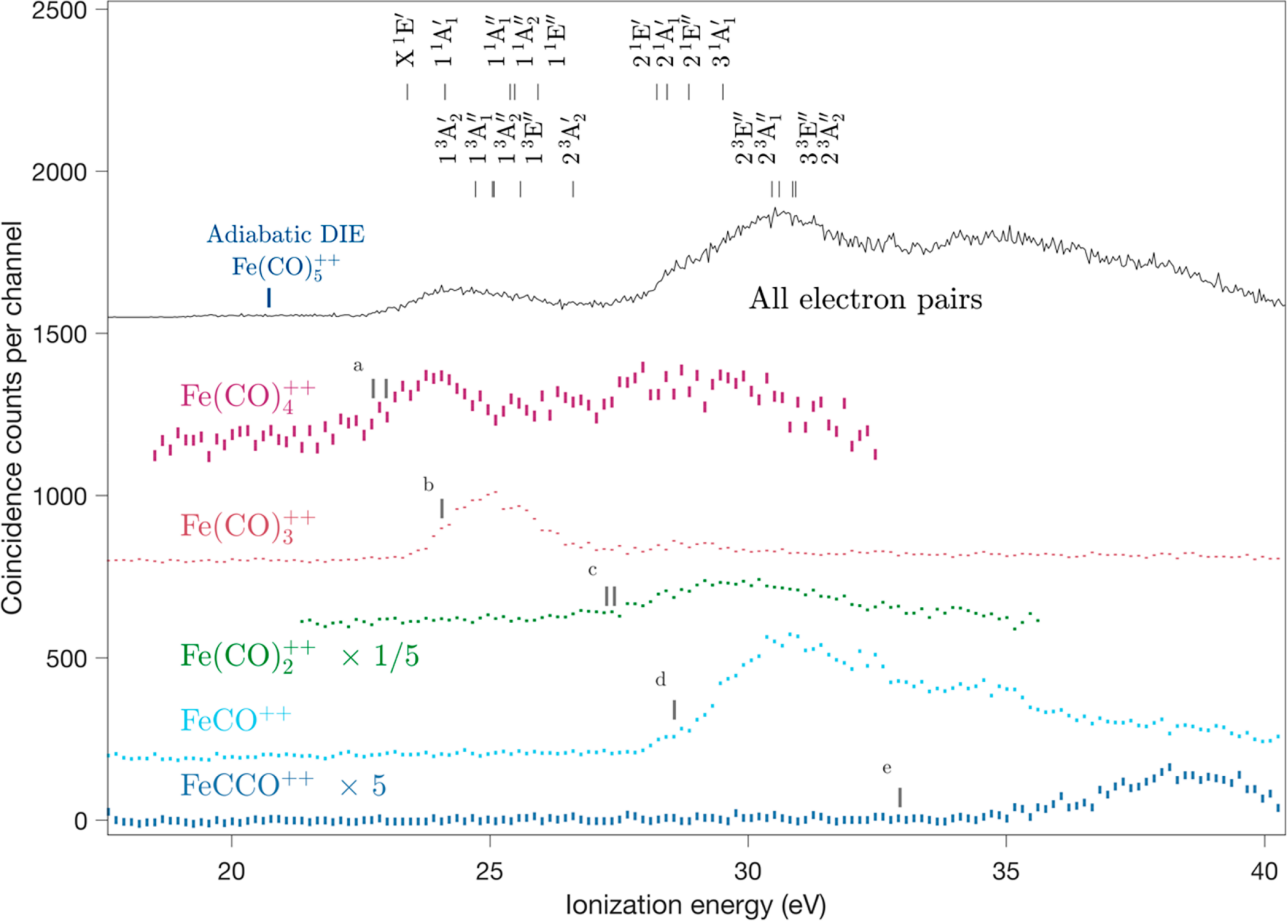
Spectra coincident with charge-retaining fragments from Fe(CO)_5_ photoionized at 40.81 eV from threefold eei coincidences, with a double ionization spectrum from electron-only experiments for comparison. Note the scales of intensity for Fe(CO)_2_^++^ (reduced) and for FeCCO^++^ (increased). The error bars at each point give statistical uncertainties only. Uncertainty in the factors for the subtractions required to produce the curves for Fe(CO)_2_^++^ and Fe(CO)_4_^++^ are not represented. Bars representing calculated vertical ionization energies of doubly ionized states are included. Appearance energies for the fragmentation channels (a) Fe(CO)_4_^++^ + CO, (b) Fe(CO)_3_^++^ + 2CO, (c) Fe(CO)_2_^++^ + 3CO, (d) Fe(CO)^++^ + 4CO, and (e) FeCCO^++^ + O + 3CO are also marked.

To obtain the spectra coincident with the formation of double-charge retaining fragments, threefold eei coincidences are sufficient if peaks at the relevant mass-to-charge ratios are not overlapped by singly charged ions. For the cases where singly and doubly charged ions do overlap, spectra of the charge-retaining components can be extracted provisionally by subtracting scaled versions of the relevant fourfold (eeii) coincidence spectra from the threefold ones. The necessary scaling factors are the composite collection and detection efficiencies for the fragment ions in question, which are known only approximately; they depend on both apparatus and ion-specific effects including the ion kinetic energies and angular distributions. As the efficiency is near 10%, scaling is by a factor of about 10, introducing serious uncertainty and amplifying the statistical spread. The spectra for the Fe(CO)_4_^++^ and Fe(CO)_2_^++^ ions, as shown in Figure 2, must therefore be treated with great caution, especially since the KER may vary as a function of the ionization energy (available excitation energy in the dissociating parent species) affecting the required scaling factor. If the KER rises with ionization energy, as expected, the collection/detection efficiency for charge separated ions will fall, and the residual doubly charged precursor intensity will be overestimated after the subtraction.

In agreement with their abundance, as suggested by the mass spectrum in Figure 1, Fe(CO)_2_^++^ ions show up with high intensity in Figure 2, surpassing Fe(CO)_3_^++^. The heaviest detected doubly charged ion, Fe(CO)_4_^++^, appears in medium abundance, starting with a broad band at the lowest accessible double ionization energies. The apparent yield of this ion above 25 eV may be an artifact of the uncertain subtraction procedure. The sum of all the charge-retaining product yields matches the shape of the total double ionization spectrum well up to about 32 eV, demonstrating that these are the dominant decay processes in the low-energy range.

**Figure 3:**
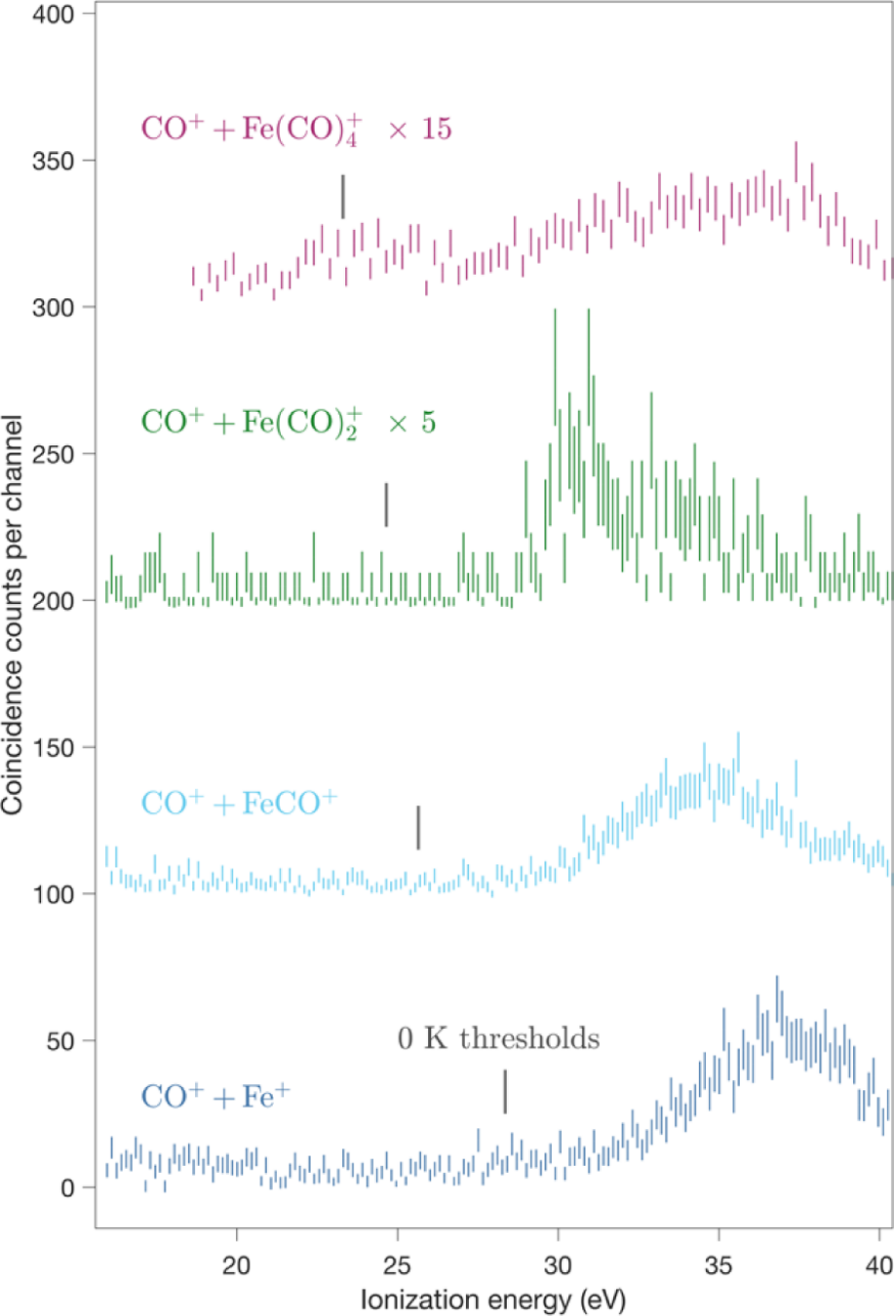
Spectra coincident with charge-separated products from the decay of Fe(CO)_5_ at 40.81 eV, from the same run as the data of Figure 2. The three lower curves are from fourfold eeii coincidences, while the uppermost one is from threefold coincidences, as explained in the text. All are on the same scale with the indicated factors, displaced vertically for clarity. Thermochemical thresholds for the formation of the products at 0 K with no KER, derived from the numerical data of Distefano^21^ are marked by vertical lines for comparison.

For charge-separating dissociation pathways, fourfold eeii coincidence measurements give spectral yield curves uncontaminated by ion overlap. Since the ion collection and detection efficiency is only about 10%, the number of counts is however low. For the least intense observed charge-separation channel, CO^+^ + Fe(CO)_4_^+^, the number of fourfold coincidence counts is too low (ca 1 count per 0.2 eV channel) to be useful. To get a spectrum, we take the threefold coincidence curve for Fe(CO)_4_^+^ as a proxy, limiting the acceptable electron energy to the double ionization energy range of 0–20 eV (21–41 eV ionization energy); this is shown in Figure 3 with the fourfold coincidence curves. The channel forming CO^+^ + Fe(CO)_3_^+^ is too weak even in threefold coincidences to yield a useful spectrum, although it is just detectable in ion–ion coincidence maps.^25^

The gaps between the thermochemical thresholds and actual onsets of significant intensity for the charge separation in Figure 3 arise mainly from KERs due to the Coulomb repulsion. The magnitudes of these charge-separation KERs, rather accurately known from the work of Hsieh and Eland,^25^ are around 3 eV, whereas each CO evaporation involves a KER of only about 0.1 eV, as determined from the measurement of the ion time-of-flight peak widths. To compare the relative intensities of competing pathways in decay of a single (assumed) precursor, the numbers of counts, as shown in Figure 3, should be multiplied by 10, or equivalently those in Figure 2 could be divided by 10.

Figures 2 and 3 show that the relative intensities of the main fragmentation channels vary quite systematically as functions of the ionization energy, or equivalently as functions of internal excitation energy. From the onset of vertical double ionization near 23 eV up to 30 eV, first two then successively three and four neutral CO molecules are evaporated leaving long-lived doubly charged fragments. Above 30 eV charge separation gives CO^+^ with Fe(CO)_2_^+^, FeCO^+^ or Fe^+^ in relative proportions 1:1:0.5 for 30–32 eV, 0.2:1:0.6 for 32–34 eV, 0.1:1:1 for 34–36 eV, 0:1:2 for 36 to 38 eV, and 0:1:2.5 for 38–40 eV. The proportions are given here explicitly as they might be useful as indirect measures of the excitation energy in work where double ionization free from single ionization is selected, such as in ion–ion coincidence experiments with ionization by particle impact. Because of the mass number overlaps mentioned at the beginning of this section, simple mass spectral peak intensities are not useful for such a purpose.

### Computational Results

**Figure 4:**
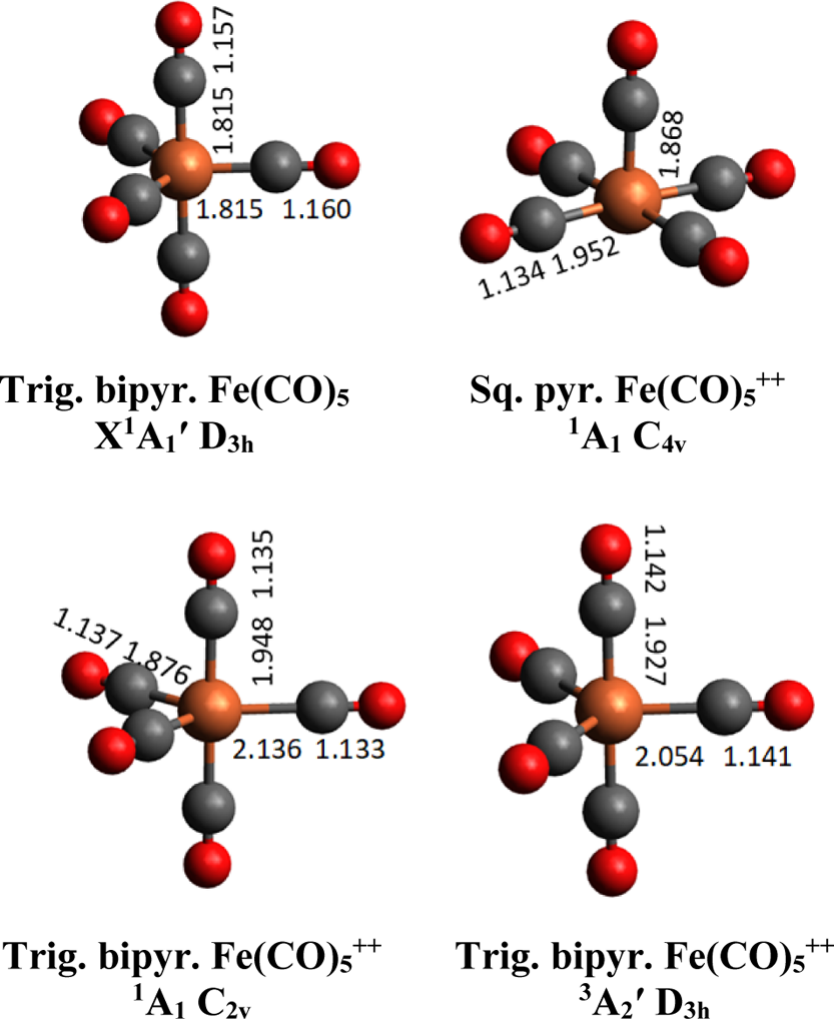
Top: stable structures of Fe(CO)_5_ and of Fe(CO)_5_^++^, as optimized at the (R)CCSD(T)/aug-cc-pVDZ-DK level of theory. Bottom: singlet and triplet transition states’ optimized structures in the Franck–Condon region accessed by vertical double photoionization of Fe(CO)_5_. The main bond distances are given in units of Ångström with the point groups and spectroscopic terms. See Table S1.

Fe(CO)_5_ has a trigonal bipyramidal structure with *D*_3*h*_ symmetry. The starting geometry for optimizations of Fe(CO)_5_ species was the structure obtained by Beagley and Schmidling.^40^Figure 4 presents the Fe(CO)_5_ and Fe(CO)_5_^2+^ ground-state stable structures, as computed at the CCSD(T)/aug-cc-pVDZ-DK level. For the neutral molecule, we obtained the expected *D*_3*h*_ trigonal bipyramid structure, whereas for the dication, a *C*_4*v*_ square pyramid structure was found. Figure 4 suggests that dicationic trigonal bipyramid singlet and triplet species correspond to transition states on their respective potential energy surface, rather than stable forms. Therefore, large distortions from *D*_3*h*_ to *C*_4*v*_ occur on doubly ionizing Fe(CO)_5_. This results in close-to-zero Franck–Condon factors for the Fe(CO)_5_ (X^1^A_1_^′^) → Fe(CO)_5_^++^ (X^1^A_1_) + 2e^–^transition. The adiabatic double ionization energy (ADIE) and the vertical double ionization energy (VDIE) should be quite different. From the present computations, we determined ADIE = 20.72 eV and VDIE = 23.40 eV, with an expected onset of a measureable double ionization signal at 22.71 eV, in agreement with the experimental spectrum where an onset at 22.9 eV is observed.^26^

Table 1 gives vertical excitation energies to electronic states of Fe(CO)_5_^++^ at the ground-state neutral equilibrium geometry calculated at the CASSCF/aug-cc-pVDZ-DK level with their dominant electron configurations. There is a high density of electronic states in the 23–31 eV energy range, which together with the expected geometry changes upon double ionization will lead to congestion of bands, as observed in the experimental spectrum (cf. Figure 2). As can be seen, the calculated state energies fall into two groups, the first extending from 23 to 27 eV corresponding to the removal of two electrons from the outermost a_2_^″^, e″, e′ molecular orbitals (MOs). The second group of states cover the range from 28 to 31 eV and are formed mainly by ejecting one electron from the a_2_^″^, e″, and e′ MOs of the iron pentacarbonyl moiety and simultaneously removing a second electron from the lowest e′ orbital are primarily associated with a CO ligand. The ranges of the two groups of states correspond closely to the first two main bands in the double ionization spectrum (cf. Figure 2). As discussed by Atkins et al.,^41^ these outermost MOs each involve the five iron 3d-orbitals combined with either σ orbitals of the carbonyl ligands with no π contributions, or pure π-orbitals. In the double ionization spectrum, there are further bands at energies above 31 eV; we expect the density of electronic states to be equally high or higher in this energy range and to involve ionization from orbitals located largely on the CO ligands.

**Table 1:** Vertical Ionization Energies from the Neutral Ground State to States of Fe(CO)_5_^++^ with the Same Geometry at the CASSCF/aug-cc-pVDZ-DK Level of Theory^a^

electronic state	electronic configuration	energy/eV
X ^1^E′	(a_2_^″^)^2^(e″)^4^(e′)^2^	23.40
1 ^1^A_1_^′^	(a_2_^″^)^2^(e″)^4^(e′)^2^	24.13
1 ^3^A_2_^′^	(a_2_^″^)^2^(e″)^4^(e′)^2^	24.72
1 ^3^A_1_^″^	(a_2_^″^)^2^(e″)^3^(e′)^3^	25.05
1 ^3^A_2_^″^	(a_2_^″^)^2^(e″)^3^(e′)^3^	25.08
1 ^1^A_1_^″^	(a_2_^″^)^2^(e″)^3^(e′)^3^	25.39
1 ^1^A_2_^″^	(a_2_^″^)^2^(e″)^3^(e′)^3^	25.48
1 ^3^E^″^	(a_2_^″^)^2^(e″)^3^(e′)^3^	25.59
1 ^1^E^″^	(a_2_^″^)^2^(e″)^3^(e′)^3^	25.93
2 ^3^A_2_^′^	(a_2_^″^)^2^(e″)^2^(e′)^4^	26.61
2 ^1^E′	(a_2_^″^)^2^(e″)^2^(e′)^4^	28.23
2 ^1^A_1_^′^	(e′)3(a_2_^″^)^2^(e″)^4^(e′)^3^	28.43
2 ^1^E″	(a_2_^″^)^1^(e″)^4^(e′)^3^	28.85
3 ^1^A_1_^′^	(a_2_^″^)^2^(e″)^2^(e′)^4^	29.51
2 ^3^E″	(a_2_^″^)^1^(e″)^4^(e′)^3^	30.46
2 ^3^A_1_^″^	(e″)^3^(a_2_^″^)^2^(e″)^4^(e′)^3^	30.60
3 ^3^E″	(e″)^3^(a_2_^″^)^2^(e″)^4^(e′)^3^	30.86
	& (e″)^4^(a_2_^″^)^1^(e″)^4^(e′)^3^	
2 ^3^A_2_^″^	(e″)^3^(a_2_^″^)^2^(e″)^4^(e′)^3^	30.93

a We give also their dominant electronic configurations quoted at the equilibiurm geometry of neutral Fe(CO)_5_ ground state, where only the outermost molecular orbitals are considered.

**Figure 5:**
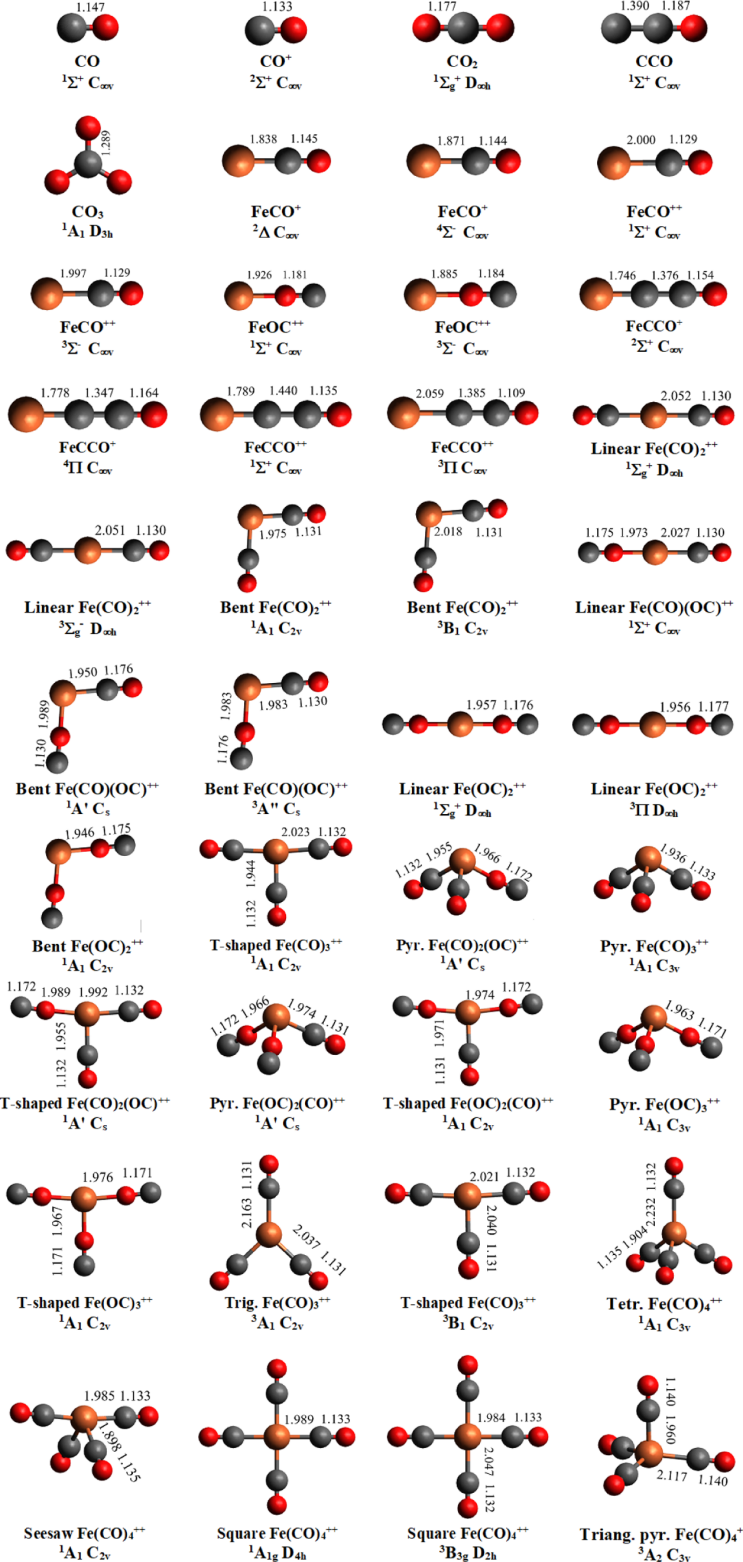
Structures of possible neutral and ionic fragments from Fe(CO)_5_^2+^ optimized at the (R)CCSD(T)/aug-cc-pVDZ-DK level of theory.

Figure 5 shows the calculated stable structures of the important fragments from dissociation of Fe(CO)_5_^++^ with their symmetries and term symbols. For some neutral, singly and doubly charged species of the same chemical formula, there are several stable isomeric forms. In addition to the expected Fe–C bonded forms, there are hitherto unknown Fe–O bonded isomers lying close in energy. In Table 2, calculated dissociation asymptotes for the charge-retaining fragments, as shown in Figure 5 are given, for comparison with the experimental appearance energies from the data of Figure 2.

**Table 2:** (R)CCSD(T)/aug-cc-pVDZ-DK Computed Dissociation Asymptotes (Δ*E*_calc_, eV), Including Zero-Point Vibrational Energies, for Charge-Retaining Fragments from Double Ionization of Fe(CO)_5_^a^

process	Δ*E*_calc_	AE_obs_
Fe(CO)_5_ → square Fe(CO)_4_^++^ (triplet) + CO	22.09	
Fe(CO)_5_ → triang pyr. Fe(CO)_4_^++^ (triplet) + CO	22.74	22.8±0.3
Fe(CO)_5_ → seesaw Fe(CO)_4_^++^ (singlet)+ CO	22.76	
Fe(CO)_5_ → square Fe(CO)_4_^++^ (singlet) + CO	22.99	
Fe(CO)_5_ → T-shaped Fe(CO)_3_^++^ (triplet) + 2CO	24.07	23.9±0.2
Fe(CO)_5_ → pyr. Fe(CO)_3_^++^ (singlet) + 2CO	24.87	
Fe(CO)_5_ → T-shaped Fe(CO)_3_^++^ (singlet) + 2CO	25.04	
Fe(CO)_5_ → pyr. Fe(CO)_2_(OC)^++^ (singlet) + 2CO	25.72	
Fe(CO)_5_ → T-shaped Fe(CO)_2_(OC)^++^ (singlet) + 2CO	25.73	
Fe(CO)_5_ → T-shaped Fe(OC)_2_(CO)^++^ (singlet) + 2CO	26.50	
Fe(CO)_5_ → pyr. Fe(OC)_2_(CO)^++^ (singlet) + 2CO	26.53	
Fe(CO)_5_ → T-shaped Fe(OC)_3_^++^ (singlet) + 2CO	27.33	
Fe(CO)_5_ → pyr. Fe(OC)_3_^++^ (singlet) + 2CO	27.34	
Fe(CO)_5_ → bent Fe(CO)_2_^++^ (triplet) + 3CO	26.24	
Fe(CO)_5_ → lin. Fe(CO)_2_^++^ (triplet) + 3CO	26.09	
Fe(CO)_5_ → bent Fe(CO) (OC)^++^ (triplet) + 3CO	26.97	
Fe(CO)_5_ → bent Fe(CO)_2_^++^ (singlet) + 3CO	27.26	27.2±0.5
Fe(CO)_5_ → lin. Fe(CO)_2_^++^ (singlet) + 3CO	27.41	
Fe(CO)_5_ → lin. Fe(OC)_2_^++^ (triplet) + 3CO	27.69	
Fe(CO)_5_ → bent Fe(CO) (OC)^++^ (singlet) + 3CO	28.11	
Fe(CO)_5_ → lin. Fe(OC)_2_^++^ (singlet) + 3CO	28.88	
Fe(CO)_5_ → bent Fe(OC)_2_^++^ (singlet) + 3CO	28.92	
Fe(CO)_5_ → Fe(CO)^+^ (quartet) + CO^+^ + 3CO	25.82	
Fe(CO)_5_ → Fe(CO)^+^ (doublet) + CO^+^ + 3CO	27.73	27.5±0.5
Fe(CO)_5_ → Fe(CO)^++^ (triplet) + 4CO	28.57	28.2±0.2
Fe(CO)_5_ → Fe(CO)^++^ (singlet) + 4CO	29.89	
Fe(CO)_5_ → Fe(OC)^++^ (singlet) + 4CO	30.77	
Fe(CO)_5_ → FeCCO^++^ (X^3^Σ^–^)+ CO_2_ + 2CO	27.42	
Fe(CO)_5_ → FeCCO^++^ (X^3^Σ^–^)+ O + 3CO	32.94	34.7±0.4
Fe(CO)_5_ → FeCCO^++^ (X^3^Σ^–^) + CO_3_ + CCO	37.01	
Fe(CO)_5_ → FeCCO^++^ (X^3^Σ^–^) + CO + O_2_ + CCO	37.20	

a Our observed appearance energies (AE_obs_, eV) for fragment ions of the corresponding masses are given for comparison. The fragments are assumed to be in their lowest electronic state of the indicated spin-multiplicity.

## Discussion

The onset of double ionization observed by our technique in Fe(CO)_5_ is 2 eV above the calculated ADIE, largely because of the change in geometry from *D*_3*h*_ to *C*_4*v*_ required to reach the adiabatic limit. Single and double photoionization at a fixed photon energy below all inner shell onsets, as practised here, are essentially vertical processes. Indirect double ionization routes such as Auger electron ejection involving singly ionized or neutral species as intermediates might allow closer approach to the adiabatic limit. Some indirect pathways are allowed in electron impact ionization at 70 eV and may account for the observation of stable or long-lived metastable Fe(CO)_5_^++^ dications in the early mass spectra^18,19^ in contrast to their absence here in one-photon double ionization.

Fragmentation of all the dicationic species, including the heaviest precursor, Fe(CO)_5_^++^, may follow two-body or multi-body decay pathways, and for any set of products, the total charge may either be retained on a single fragment or perhaps end up as separate single charges on two fragments. The parent dication could undergo competing decays by two possible two-body pathways
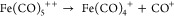
1
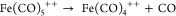
2

Whether or not these pathways can actually compete with each other depends on their energy requirements. For the charge separation, the known thermochemistry, based on the measurements of Distefano^21^ gives an asymptotic limit of 22.78 eV for the formation of the products of reaction 1 in their ground states with no KER. Coulomb repulsion between the two separated charges will result in a KER of about 3 eV, bringing the predicted actual appearance energy (AE) to above 25 eV, in good agreement with our observation, as shown in Figure 3. In contrast, both the present calculations and experiment show that the AE for charge retention by Fe(CO)_4_^++^ is near 23 eV; this means that in the energy range of 23–25 eV, charge retention is the only energetically allowed pathway. The complications do, however, not end there. Although we can exclude the formation of square-planar Fe(CO)_4_^++^ in the triplet state, the calculations in Table 2 show that at least three isomeric forms of Fe(CO)_4_^++^ (triangular pyramidal ions as triplets and “seesaw” and square-planar Fe(CO)_4_^++^ as singlets) have AEs indistinguishable from the experimentally determined value. In general, because of this high density of states, we expect internal conversions and intersystem crossings to efficiently couple all excitations to the lowest state. Where big changes of molecular geometry occur, substantial vibrational excitation is expected too, but as the vibrational frequencies relating to CO repositioning are low, these probably do not affect the energetics appreciably.

For the lighter charge-retaining fragments, multiple formation pathways are possible. For the ions of formula FeC_3_O_3_^++^, for instance, there could be
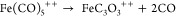
or
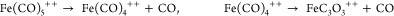


The choice between three-body and two-body-sequential pathways does not change the energy requirements, and for every dicationic fragment, there is a range of energies where they can be formed without competition from charge separation. This is an unusual situation, which arises because of the relatively high ionization energy of CO and the low double ionization energies of the Fe-containing fragments.

For the related charge separations possible paths are
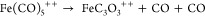
or
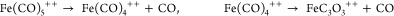


In this and all the similar cases, the momentum correlations investigated by Hsieh and Eland^25^ clearly show that the sequential paths are dominant. Of the possible structures of the FeC_3_O_3_^2+^ dication, comparison between the experimental and computed AEs (Table 2) suggests that the T-shaped Fe(CO)_3_^++^ in its triplet state is the form actually produced, at least near threshold as the computed (24.07 eV) and measured (23.9 ± 0.2 eV) thresholds for the Fe(CO)_5_^++^ → FeC_3_O_3_^++^ + 2CO reaction coincide within the error limits of the calculations and experiments. We note that axial and equatorial CO ligands are not equivalent in neutral Fe(CO)_5_, and the T-shaped form can be created most economically by loss of two axial CO moieties.

For FeC_2_O_2_^++^, ions of two structures in singlet states are within the range of the experimental AE, namely, bent and linear Fe(CO)_2_^++^, but three other isomeric forms, all with some Fe–O bonding, may contribute at higher ionization energies. These are bent Fe(CO)(OC)^2+^ (singlet), linear Fe(OC)_2_^2+^ (triplet), and bent Fe(OC)_2_^2+^ (singlet). For FeCO^++^, the calculated appearance energy of the normal Fe–C bonded form in the triplet state agrees well with the experimental AE, while the singlet of the same form and the FeOC^++^ form are excluded, at least near threshold.

In addition to the main dissociation pathways, the mass spectra and ion–ion coincidence measurements^25^ show that there are minor decay pathways producing the rather more exotic ions FeC^+^, FeO^+^, FeCCO^+^, and FeCCO^++^ from initial double ionization. Of these, we have concentrated exclusively on the FeCCO^++^ dication, whose formation at an onset near 35 eV (cf. Figure 2), compared with the calculated appearance energy of 32.94 eV, proves that the accompanying products are atomic O and three CO molecules, in agreement with the proposal of Lacko et al.^42^ The linear FeCCO^++^ dication is formed in its ground triplet state, and other possible combinations of products are excluded (see Table 2). We note that at 40.81 eV photon energy and starting at an ionization energy near 36 eV, a free CO molecule undergoes dissociative double ionization,^26^ initially forming C^+^ + O*. The superexcited O* atom subsequently autoionizes to O^+^. If the same initial process happens in the context of an Fe(CO)_5_ molecule, it is reasonable to postulate that the C^+^ ion, already adjacent to the Fe atom, may cleave to it, and the O^+^ may exchange its charge to the larger (lower IE) molecular framework and escape as a neutral atom. Rapid evaporation of three more CO ligands could then follow. Our calculations show that the FeCCO^++^ ion has the cumulenic structure, Fe=C=C=O, perfectly compatible with the above hypothesis. Our computed Fe–C bond distance is close to the computed values found by Pu et al.^43^ for the related cumulenic structures [FeC_*m*_Fe]^++^. Although this reaction is observed at high energy in the gas phase, we speculate that a similar mechanism might be relevant in clusters or in solution where ions are highly stabilized relative to their free gas phase forms.

For the charge separating reactions, thermochemical thresholds are available by adding the well-known ionization energy of CO (14.01 eV) to the AEs of the singly charged Fe(CO)_*n*_^+^ ions determined by Distefano.^21^ As seen in Figure 3, experimentally the onsets of all four observed ion pairs are gradual, making it impossible to determine precise AEs. All the ion pair yields start several electron volts above the thermochemical onsets because of KERs from Coulomb repulsion in charge separation. Asymptotic limits obtained in this way assume, of course, that the Fe(CO)_*n*_^+^ product ions are formed in the same states at threshold by single and double ionization. As one check on this and to identify the ion state, we have calculated possible asymptotes for FeCO^+^ + CO^+^ + 3CO, where the thermochemical value is 25.81 eV. For the lowest quartet state of FeCO^+^, the calculated asymptote is 25.83 eV, in very good agreement, whereas for the doublet state, 27.73 eV is calculated. For this pair, the yield curve rises perceptibly above background at about 29 eV, suggesting a total energy release (kinetic + internal) of 3.5 eV. The measured KER is close to 3.0 ± 0.1 eV, with the exact value dependent on the method of its determination from the peak shape.^25^

If the whole spectrum, as given in Figure 2, for Fe(CO)_4_^++^ is valid (despite the uncertainty consequent upon subtraction of the dominant monocation signal at the same mass number), charge retention and charge separation may be in competition for Fe(CO)_5_^++^ over a range of energies. For the lighter dications Fe(CO)_4_^++^, Fe(CO)_3_^++^, and Fe(CO)_2_^++^, however, comparison between Figures 2 and 3 shows that once energetically allowed, charge separation dominates over charge retention. For FeCO^++^, the case is less clear, and competition may continue over several electronvolts.

For all the doubly charged ions, even in the range where charge separation is energetically forbidden, charge-retaining decay by CO evaporation is possible. The characteristic lifetime for this process in Fe(CO)_5_^++^ must be very short (sub nanosecond) because the parent ion is not detected, not even as a metastable. The lighter dications, Fe(CO)_*n*_^++^ (*n* = 1 to 4), which are seen in the mass spectra, clearly have some long-lived (τ > μs) levels. If the metastable ions seen by Winters and Collins^19^ are indeed formed by unimolecular (not collision-induced) processes, then the Fe(CO)_4_^++^ and Fe(CO)_3_^++^ ions must also possess some levels with intermediate lifetimes (ns−μs) before CO evaporation.

Because of the high density of electronic and vibronic states in the species studied, we do not expect to see electronic state-specific behavior in the relationship between the overall double ionization spectrum and the different decay channels. At the lowest accessible ionization energies, formation of Fe(CO)_4_^++^ is dominant. Then, Fe(CO)_3_^++^ becomes stronger by the middle of the first double ionization band (25.0 eV). By 29 eV, Fe(CO)_2_^++^ is strongest, then starting at 30 eV and dominantly by 35 eV, FeCO^++^ takes over. At this (35 eV IE) point, where simplistic analysis would suggest ionization mainly from the CO ligands, there are about 200 counts per channel in the formation of FeCO^++^, and only about 40 counts per channel in the charge-separating pathway #1, both reported as threefold coincidences. Therefore, decay from nascent Fe(CO)_5_^++^ is mainly by neutral CO evaporation, not by charge separation, even where both are possible, regardless of the initial electronic state of the parent dication.

For the major dissociation products, a system of sequential decay pathways is suggested by the classical mass-spectral metastable decay reactions observed under 70 eV electron impact by Winters and Collins^19^ and supported by the coincidence measurements with position-sensitive ion detection by Hsieh and Eland.^25^ Winters and Collins saw no metastable ion signals for Fe(CO)_5_^++^ → Fe(CO)_4_^++^ + CO, but relatively strong ones for Fe(CO)_4_^++^ → Fe(CO)_3_^++^ + CO, Fe(CO)_3_^++^ → Fe(CO)_2_^++^ + CO, and Fe(CO)_2_^++^ → FeCO^++^ + CO. It follows that CO evaporation is the major initial process, with charge separations happening only after formation of the different dications, and at a slower pace. Our data show that once the dications have shed two or more CO molecules, charge separations become more competitive. This general scheme is also supported by the breakdown pattern implied by Figures 2 and 3, most clearly for the lighter dications. The yield of FeCO^+^ + CO^+^ rises in the range 30–35 eV, just as the yield of Fe(CO)_2_^++^ falls. Similarly, the yield of Fe^+^ + CO^+^ rises in the range 31–37 eV as that of FeCO^++^ diminishes. The complementarity of the rises and falls is essentially quantitative in counts as fractions of total ionization when the relevant collection/detection efficiencies are allowed for.

From the data in Figures 2 and 3, we can extract appearance energies for comparison with other experiments and with our theoretical results. For the channels with gradual onsets, or onsets confused by poor statistics, the error limits are necessarily wide (Table 3).

**Table 3:** Summary of Appearance Energies (eV)

ionic products	this work			
	exp.	calc.^f^	Lacko et al.^a^	Conard et al.^b^
Fe(CO)_4_^++^	22.8 ± 0.2	22.74–22.99	22.74	
Fe(CO)_3_^++^	23.7 ± 0.2	24.07	23.57	24.0
Fe(CO)_2_^++^	26.8–27.5	27.26, 27.41		
FeCCO^++^	34.7 ± 0.4	32.94	36.36	
FeCO^++^	28.2 ± 0.2	28.57	30.06	
Fe(CO)_2_^+^ + CO^+^	29.5 ± 0.3	*24.6* 9 + KER		
FeCO^+^ + CO^+^	30 ± 0.5	25.83 + KER		
Fe^+^ + CO^+^	31 ± 0.5	*28.04* + KER		
Fe(CO)_5_^++^	c	20.72^d^		
23.4^e^				

a Ref (42).

b Ref (44).

c Not detected. Double ionization starts 22.7 ± 0.3 eV.

d ADIE.

e VDIE.

f Where we have not calculated the asymptote, the thermochemical value is listed (italics). Not listed are the thermochemical asymptotes for Fe(CO)_4_^+^ + CO^+^ (22.80 eV) and Fe(CO)_3_^+^ + CO^+^ (23.88 eV).

## Conclusions

Using threefold and fourfold electron–ion coincidence measurements together with high-level calculations, we have characterized the energetics and breakdown pathways of doubly ionized iron pentacarbonyl in quite some detail. The parent dication’s most stable state has a quite different structure from that of the neutral molecule and is not accessed by vertical double ionization. Alternative energetically possible structures also exist for several of the doubly charged fragments. The dominant dissociation pathway is evaporation of one to four CO molecules in succession, the number lost increasing as the ionization energy gets higher. After each stage of CO loss, and as the ionization energy rises, the final stage of each decay chain is the emission of a CO^+^ ion. The formation of new Fe–C and C–C bonds occurs in the dications only at high ionization energies, a finding which is likely to be relevant to catalysis by Fe.

## Supporting Information Available

Optimized structures, energies, harmonic frequencies, and spectroscopic terms of iron carbonyls and related ions and fragments (PDF)
