# Supplementary material for: Unimolecular Double Photoionization-Induced Processes in Iron Pentacarbonyl
Source: Inorg Chem. 2021 Oct 26;60(23):17966–75. doi: 10.1021/acs.inorgchem.1c02533 (PMC8653154; doi:10.1021/acs.inorgchem.1c02533)
Supplement: Supplementary file 1 — ic1c02533_si_001.pdf [file ic1c02533_si_001.pdf]

Supporting Information

Unimolecular double photoionization-induced processes in iron pentacarbonyl

Roberto Linguerri[a], Emelie Olsson[b], Gunnar Nyman[c], Majdi Hochlaf[a*], John H.D. Eland[d*] and Raimund Feifel[b*]

[a] *Université Gustave Eiffel, COSYS/LISIS, 5 Bd Descartes 77454, Champs sur Marne, France.*
[b] *University of Gothenburg, Department of Physics, Origovägen 6B, 412 58 Gothenburg, Sweden.*
[c] *University of Gothenburg, Department of Chemistry and Molecular Biology, 405 30 Gothenburg, Sweden.*
[d] *Oxford University, Department of Chemistry, Physical and Theoretical Chemistry Laboratory, South Parks Road, Oxford OX1 3QZ, United Kingdom.*
[*] *Corresponding authors: majdi.hochlaf@univ-eiffel.fr; john.eland@chem.ox.ac.uk; raimund.feifel@physics.gu.se.*

Table S1: Optimized structures of molecular species of interest as computed at the (R)CCSD(T) /aug-cc-pVDZ-DK level. We give their total energies, harmonic frequencies, spectroscopic terms and their bond distances (in Å).

	Molecular geometry with main distances in Å.	Electronic state term	Energy (Hartree)	Harmonic frequencies (cm^{-1})
CO	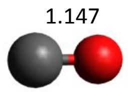	$^{1}\Sigma^{+}$	-113.14081991	2102.6(σ^{+})
CO^{+}	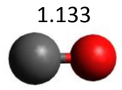	$^{2}\Sigma^{+}$	-112.63382070	2152.0(σ^{+})
$FeCO^{+}$	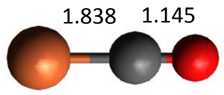	$^{2}\Delta$	-1384.32821200	355.5(π)<br>421.9(σ^{+})<br>2071.7(σ^{+})
$FeCO^{2+}$	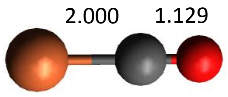	$^{1}\Sigma^{+}$	-1383.74114884	308.8(π)<br>389.3(σ^{+})<br>2163.5(σ^{+})
$FeOC^{2+}$	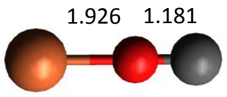	$^{1}\Sigma^{+}$	-1383.70791753	170.3(π)<br>413.2(σ^{+})<br>1908.7(σ^{+})
Linear $Fe(CO)_2^{2+}$	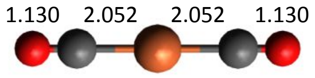	$^{1}\Sigma_g^{+}$	-1496.97686473	93.8(π_u)<br>297.2(π_g)<br>311.0(σ_g^{+})<br>405.9(σ_u^{+})<br>408.4(π_u)<br>2157.7(σ_u^{+})<br>2158.1(σ_g^{+})
Bent $Fe(CO)_2^{2+}$	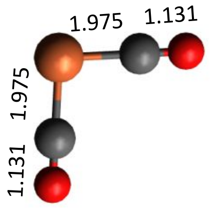	$^{1}A_1$	-1496.98195615	88.8(a_1)<br>301.7(a_2)<br>303.7(b_2)<br>309.3(b_1)<br>330.0(a_1)<br>386.1(b_2)<br>449.0(a_1)<br>2150.7(a_1)<br>2157.9(b_2)
Linear $Fe(CO)(OC)^{2+}$	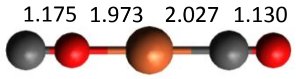	$^{1}\Sigma^{+}$	-1496.94981334	87.2(π)<br>189.8(π)<br>313.8(σ^{+})<br>352.1(π)<br>424.0(σ^{+})<br>1938.6(σ^{+})<br>2165.0(σ^{+})

Bent $Fe(CO)(OC)^{2+}$	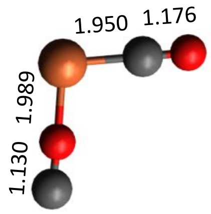	$^{1}A'$	-1496.95037783	81.7(a′)<br>167.1(a″)<br>184.4(a′)<br>306.2(a″)<br>332.7(a′)<br>384.4(a′)<br>392.3(a′)<br>1938.0(a′)<br>2167.5(a′)
Linear $Fe(OC)_2^{2+}$	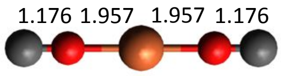	$^{1}\Sigma_g^+$	-1496.92024325	89.4(π_u)<br>174.6(π_g)<br>224.1(π_u)<br>325.7(σ_g^+)<br>439.1(σ_u^+)<br>1927.2(σ_u^+)<br>1943.0(σ_g^+)
Bent $Fe(OC)_2^{2+}$	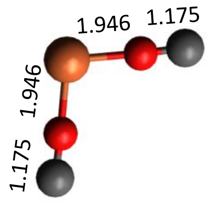	$^{1}A_1$	-1496.91865052	81.9(a_1)<br>167.8(a_2)<br>168.3(b_2)<br>172.3(b_1)<br>211.6(a_1)<br>383.5(a_1)<br>395.2(b_2)<br>1928.4(b_2)<br>1941.8(a_1)
Pyr. $Fe(CO)_3^{2+}$	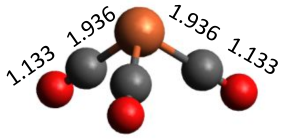	$^{1}A_1$	-1610.21393892	81.87(e)<br>96.76(a_1)<br>297.47(a_1)<br>306.09(e)<br>308.95(a_2)<br>358.00(e)<br>456.33(e)<br>485.01(a_1)<br>2131.86(a_1)<br>2143.35(e)
T-shaped $Fe(CO)_3^{2+}$	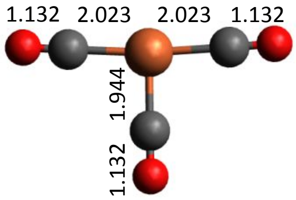	$^{1}A_1$	-1610.20752549	85.1<br>86.4<br>93.7<br>287.6<br>292.8<br>302.4<br>307.5<br>349.8<br>367.9<br>419.3<br>454.9<br>481.3<br>2138.9<br>2144.3<br>2150.1
Pyr. $Fe(CO)_2(OC)^{2+}$	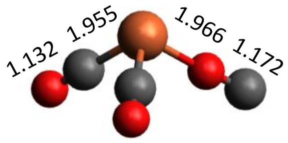	$^{1}A'$	-1610.18147184	81.0<br>84.1<br>96.1<br>183.6<br>185.7<br>305.4<br>310.6<br>338.7<br>344.3<br>387.1

	1.132			392.3<br>457.7<br>1958.6<br>2151.5<br>2158.8
T-shaped $Fe(CO)_2(OC)^{2+}$	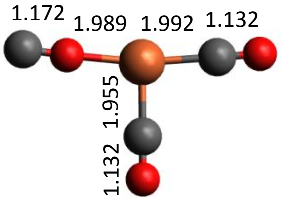	$^1A'$	-1610.18080955	85.2<br>87.7<br>93.1<br>184.6<br>191.1<br>289.0<br>307.5<br>336.5<br>358.3<br>358.7<br>401.1<br>471.6<br>1957.0<br>2150.4<br>2159.9
Pyr. $Fe(OC)_2(CO)^{2+}$	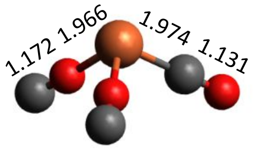	$^1A'$	-1610.15008635	75.1<br>80.2<br>90.0<br>164.3<br>180.7<br>188.8<br>208.5<br>321.9<br>334.5<br>376.8<br>382.1<br>385.9<br>1952.0<br>1961.8<br>2168.7
T-shaped $Fe(OC)_2(CO)^{2+}$	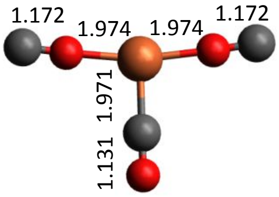	$^1A_1$	-1610.15126796	83.3<br>84.9<br>86.7<br>165.1<br>175.3<br>214.4<br>218.6<br>296.1<br>312.5<br>363.1<br>374.3<br>426.3<br>1950.3<br>1961.8<br>2171.1
Pyr. $Fe(OC)_3^{2+}$	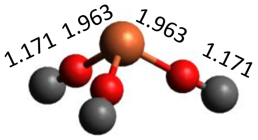	$^1A_1$	-1610.11930001	66.0<br>69.0<br>80.3<br>160.4<br>162.3<br>162.9<br>206.0<br>207.2<br>213.8<br>359.9

				373.4<br>374.2<br>1948.6<br>1948.7<br>1963.5
T-shaped $Fe(OC)_3^{2+}$	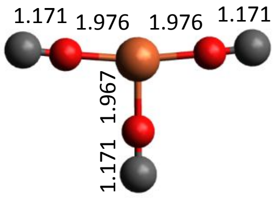	$^1A_1$	-1610.11954313	83.7<br>84.1<br>84.3<br>163.9<br>164.3<br>167.9<br>219.7<br>220.2<br>221.2<br>305.2<br>367.1<br>414.6<br>1947.0<br>1951.5<br>1966.2
Seesaw $Fe(CO)_4^{2+}$	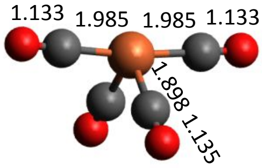	$^1A_1$	-1723.43587223	68.5<br>79.7<br>91.7<br>97.1<br>100.7<br>268.7<br>314.2<br>315.2<br>324.7<br>343.1<br>357.1<br>365.9<br>446.9<br>483.3<br>497.9<br>529.3<br>536.4<br>2110.5<br>2124.6<br>2125.4<br>2133.0
Square $Fe(CO)_4^{2+}$	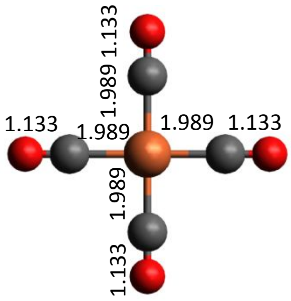	$^1A_{1g}$	-1723.42755261	68.8<br>101.5<br>104.6<br>106.3<br>118.7<br>282.8<br>311.1<br>311.1<br>322.5<br>333.4<br>377.1<br>377.3<br>415.0<br>498.5<br>500.6<br>531.5<br>531.6<br>2125.0<br>2130.9

				2130.9<br>2141.1
Tetr. $Fe(CO)_4^{2+}$	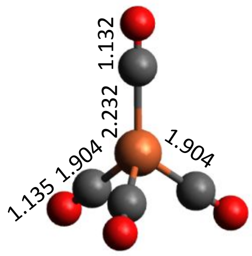	$^1A_1$	-1723.41354977	*i* 127.7<br>*i* 124.4<br>89.9<br>93.0<br>103.7<br>190.6<br>191.5<br>228.3<br>305.0<br>305.9<br>321.5<br>337.3<br>368.3<br>368.6<br>494.1<br>494.3<br>537.9<br>2115.7<br>2128.9<br>2131.2<br>2131.5
Trig. bipyr.<br>$Fe(CO)_5$	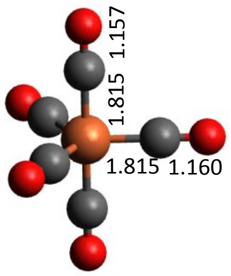	$^1A'_1$	-1837.42459499	66.5<br>83.7<br>91.9<br>110.6<br>120.2<br>154.3<br>155.0<br>421.5<br>500.0<br>509.3<br>510.2<br>577.8<br>579.1<br>595.5<br>597.4<br>604.2<br>647.5<br>655.7<br>656.2<br>801.7<br>939.8<br>939.8<br>1941.3<br>2015.5<br>2056.9<br>2269.1<br>2269.5
Trig. bipyr.<br>$Fe(CO)_5^{2+}$	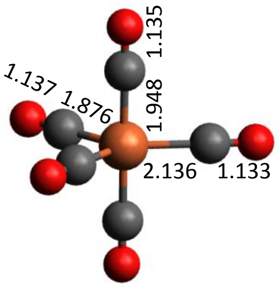	$^1A_1$	-1836.63713124	*i* 227.1<br>47.8<br>68.6<br>84.5<br>99.0<br>107.1<br>107.1<br>158.8<br>225.5<br>321.6

				334.8<br>347.8<br>347.8<br>378.4<br>382.4<br>411.6<br>472.8<br>529.7<br>539.4<br>561.2<br>611.7<br>643.9<br>2067.9<br>2087.6<br>2097.9<br>2106.9<br>2123.8
Sq. pyr. $Fe(CO)_5^{2+}$	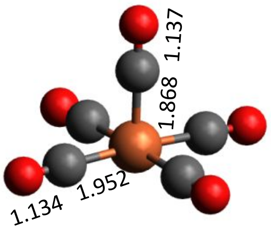	$^1A_1$	-1836.65686416	79.9<br>99.7<br>100.7<br>104.1<br>121.4<br>121.5<br>125.0<br>286.7<br>354.4<br>354.8<br>359.8<br>375.2<br>390.5<br>391.4<br>403.4<br>489.2<br>535.2<br>535.5<br>549.2<br>615.2<br>629.2<br>629.5<br>2075.2<br>2097.4<br>2097.5<br>2102.6<br>2115.3
Trig. $Fe(CO)_3^{2+}$	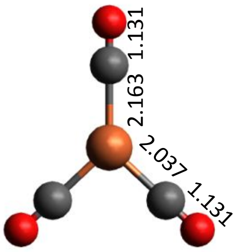	$^3A_1$	-1610.21122526	*i* 97.3<br>7.4<br>73.6<br>222.0<br>255.8<br>264.1<br>265.0<br>280.1<br>330.2<br>394.4<br>423.0<br>508.9<br>2137.6<br>2144.2<br>2154.4

T-shaped $Fe(CO)_3^{2+}$	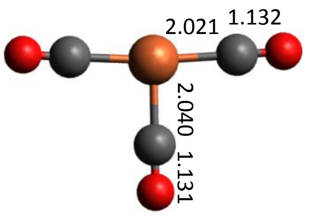	$^3B_1$	-1610.24290625	98.4<br>98.7<br>100.8<br>286.8<br>289.2<br>297.8<br>301.4<br>331.4<br>366.7<br>420.1<br>449.7<br>460.9<br>2141.6<br>2146.6<br>2152.1
Square $Fe(CO)_4^{2+}$	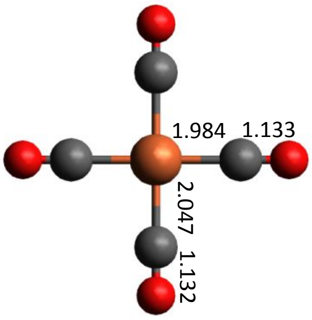	$^3B_{3g}$	-1723.45956329	*i* 11.0<br>61.6<br>83.7<br>93.0<br>98.7<br>254.1<br>284.1<br>302.3<br>304.4<br>319.8<br>321.8<br>363.5<br>369.3<br>452.0<br>483.1<br>491.9<br>521.8<br>2126.2<br>2128.8<br>2139.8<br>2143.5
Triang. pyr. $Fe(CO)_4^{2+}$	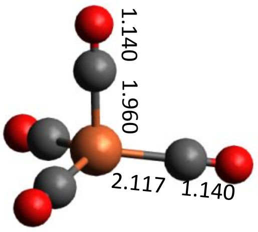	$^3A_2$	-1723.34808838	32.6<br>35.3<br>76.5<br>81.4<br>82.0<br>243.8<br>261.9<br>266.0<br>266.4<br>278.1<br>278.2<br>301.7<br>301.9<br>303.2<br>394.8<br>409.5<br>409.6<br>2131.9<br>2138.2<br>2138.3<br>2142.3

Trig. bipyr. $Fe(CO)_5^{2+}$	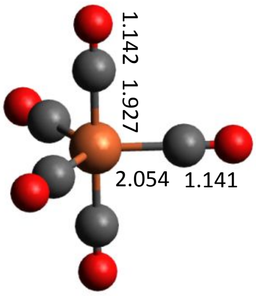	$^3A_2'$	-1836.54568321	*i* 35.4<br>*i* 20.6<br>78.8<br>88.1<br>89.8<br>98.4<br>98.6<br>222.4<br>265.2<br>272.7<br>272.9<br>300.4<br>301.2<br>315.0<br>316.6<br>318.2<br>353.9<br>461.5<br>462.4<br>523.0<br>523.1<br>526.8<br>2111.4<br>2117.7<br>2126.7<br>2127.0<br>2130.0
